# Dual-Plane Retro-pectoral Versus Pre-pectoral DTI Breast Reconstruction: An Italian Multicenter Experience

**DOI:** 10.1007/s00266-020-01892-y

**Published:** 2020-08-28

**Authors:** Diego Ribuffo, Giorgio Berna, Roy De Vita, Giovanni Di Benedetto, Emanuele Cigna, Manfredi Greco, Luigi Valdatta, Maria Giuseppina Onesti, Federico Lo Torto, Marco Marcasciano, Ugo Redi, Vittorio Quercia, Juste Kaciulyte, Mario Cherubino, Luigi Losco, Francesco Luca Rocco Mori, Alessandro Scalise

**Affiliations:** 1 Plastic Surgery Unit, Department of Surgery “Pietro Valdoni”, Sapienza University of Rome, Viale Regina Margherita 302, 00198 Rome, Italy; 2 Department of Plastic and Reconstructive Surgery, Ulss 9 General Hospital, Treviso, Italy; 3 Department of Plastic and Reconstructive Surgery, Instituti Fisioterapici Ospitalieri, Regina Elena National Cancer Institute, Rome, Italy; 4 Clinic of Plastic and Reconstructive Surgery, Department of Experimental and Clinical Medicine, Marche Polytechnic University, Via Conca 71, 60126 Ancona, Italy; 5 Department of Translational Research and New Technologies in Medicine and Surgery, University of Pisa, Pisa, Italy; 6 Plastic and Reconstructive Surgery Unit, Magna Graecia University of Catanzaro, Catanzaro, Italy; 7 Section of Medical and Surgical Sciences, Department of Biotechnology and Life Sciences, University of Insubria, 21100 Varese, Italy

**Keywords:** Pre-pectoral, ADM, Breast reconstruction, Direct-to-implant, Dual plane

## Abstract

**Background:**

The use of conservative mastectomies has risen significantly during the last few years. The reconstructive choice of direct-to-implant reconstruction has become more practicable with modern mastectomy techniques. The initial trend in Italian centers was to use dual-plane hybrid reconstruction. However, a high level of complications has been registered. From 2015 onward, in our centers, a pre-pectoral approach has been adopted. The authors sought to describe the Italian trend to gradually discard the sub-pectoral technique with lower lateral pole coverage of the prosthesis using ADMs comparing it with the pre-pectoral approach with ADMs, without any muscle dissection, in terms of complication rates.

**Materials and Methods:**

A multicenter retrospective clinical study was performed from January 2010 to June 2018. The enrolled patients were divided into two groups: Cases with an ADM-only coverage pre-pectoral reconstruction made up the first group (Group 1). Those with the retro-pectoral muscular position + ADM implant coverage comprised the second one (Group 2). Complications such as seroma, hematoma, wound dehiscence, surgical site infection, reconstruction failure, animation deformity and capsular contracture were recorded.

**Results:**

We performed 716 direct-to-implant reconstructions: 509 were partially sub-pectoral and 207 were pre-pectoral. Minimum follow-up was 1 year. Incidence of complications was higher in dual-plane reconstructions. There were statistical significant differences in the rates of seroma and hematoma.

**Conclusion:**

Using the pre-pectoral approach, the authors have experienced favorable aesthetics and superior clinical and functional outcomes. Retro-pectoral muscular ADM implant coverage has to be considered only in specific complicated second-stage surgeries.

**Level of Evidence V:**

This journal requires that authors assign a level of evidence to each article. For a full description of these Evidence-Based Medicine ratings, please refer to the Table of Contents or the online Instructions to Authors www.springer.com/00266.

**Electronic supplementary material:**

The online version of this article (10.1007/s00266-020-01892-y) contains supplementary material, which is available to authorized users.

## Introduction

When correctly indicated, conservative mastectomies (nipple sparing or skin sparing) and implant-based breast reconstruction (IBR) are safe procedures from an oncological viewpoint with satisfying aesthetic outcomes that have changed perspectives and possibilities of breast reconstruction [1]. For decades, plastic surgery writing has been dominated by the so-called two-stage reconstruction, which consists in the placement of a tissue expander in the sub-muscular space and the following replacement with a definitive implant [2].

Many alternative solutions have been developed in the past years, ranging from biological acellular dermal matrices (ADM) to meshes of various prosthetic materials. Introduction of ADMs for lower pole coverage led surgeons to perform reconstructive procedures with permanent implant without the need of expansion, the so-called direct-to-implant breast reconstruction (DTI) [3, 4].

The psychological impact of mastectomy, psychosocial distress, body image disruptions and unfavorable effects on sexual well-being are reduced to a minimum by one-stage procedures, as the breast mound is restored during the same operative episode, which also preserves good aesthetic results from total implant coverage [5].

ADMs have been used for many years as well as prosthetic meshes are approved both in Europe and in the USA. A large amount of data are present in the literature on their use and results [3, 6–14].

Due to the high rate of complications, from 2015 onward, in our centers, the dual-plane approach has been progressively limited and a pre-pectoral one has been adopted on account of the growing interest in the literature.

We aimed to describe the experience with pre-pectoral breast reconstruction we achieved after several years of clinical practice. A non-randomized retrospective study was designed in 7 Italian breast-dedicated centers. The goal of this study was to compare two surgical strategies for immediate DTI breast reconstruction with the use of ADMs. Complication rates of the sub-pectoral technique with lower lateral pole coverage of the implant with ADM were compared to the outcomes reached with pre-pectoral approach.

## Materials and Methods

A multicenter retrospective clinical study was performed from January 2010 to June 2018. We evaluated patients treated for breast cancer in 7 Italian plastic surgery units (Policlinico Umberto I, University Hospital, Rome; IFO—“Regina Elena” National Cancer Institute, Rome; S. Chiara University Hospital, Pisa; University Hospital, Ospedali Riuniti, Ancona; Mater Domini University Hospital, Catanzaro; Ulss 9, General Hospital, Treviso; University of Insubria Circolo Hospital- Fondazione Macchi, Varese). The study was conducted in accordance with the Helsinki Declaration of 1964 (revised 2008).

We were able to recognize 74 patients (35 in Group 1, 39 in Group 2) for bilateral, either therapeutic or prophylactic conservative mastectomy and 568 patients (137 in Group 1, 431 in Group 2) with cancer who received unilateral therapeutic conservative mastectomy. The mean age of the women was 56.07 years (range, 23–65 years); 95.02% of the patients were Caucasian. Patient characteristics between the two groups were well balanced, without statistically significant variance in age or BMI (Table 1).

**Table 1:** Patient demographics

Characteristic	Pre-pectoral	Dual-plane	Total	*P* value
No. of breast	207	509	716	
No of patients	172	470	642	
Mean age ± DS	55.72 ± 4.5	56.20 ± 7.6	56.07	0.8476
Mean BMI ± DS	25.36 ± 2.69	24.60 ± 3.85	24.80	0.5493
Race				
Caucasian	165 (95.93%)	445 (94.68%)	610 (95.02%)	0.6825
Other	7 (4.07%)	25 (5.32%)	32 (4.98%)	
Smoking				
Current	0	0	0	
Former	20 (11.63%)	66 (14.04%)	86 (13.40%)	0.5131
Never	152 (88.37%)	404 (85.96%)	556 (86.60%)	

Proper written informed consent was obtained from all patients. Patient demographic and characteristic data were recorded, including age at surgery, body mass index (BMI), history of smoking, breast irradiation, chemotherapy, laterality (bilateral vs unilateral). The patients who were enrolled were separated into two groups: cases with an ADM-only coverage pre-pectoral reconstruction made up the first group (Group 1). Those with the retro-pectoral muscular position + ADM implant coverage (with a biological mesh employed as a hammock to cover the lower lateral pole of the implant) comprised the second one (Group 2).

Patients were only included in the study if the subsequent criteria of selection were met: small–medium-sized breasts and ptosis grade of the first and second degree according to the three-tier Regnault ptosis scale [15]. The exclusion criteria were as follows: patients with a BMI > 30, age > 65 years, active smoking, previous breast surgery, comorbid conditions such as uncontrolled diabetes, immunogenic disorders, congestive heart failure, cardiovascular diseases including hypertension, pulmonary diseases, chronic hepatic diseases and previous radiotherapy. Former smokers were considered those who had stopped smoking at least 2 years prior to surgery.

Photographs were taken before surgery and at follow-up visits 1, 2 and 6 months postoperatively and every year thereafter to evaluate cosmetic results and assess outcomes. Complications such as seroma, hematoma, wound dehiscence, surgical-site infection, reconstruction failure (e.g., implant removal), capsular contracture and presence of animation deformity were recorded.

Wound dehiscence was defined as a disruption of sutured tissue. Signs such as erythema, pain, edema and malodorous secretions were considered suggestive of surgical site infection. In the latter case, a sample was collected and sent for culture and antibiogram in order to treat the infection with appropriate, targeted antibiotic therapy.

The determination methods used to evaluate capsular contracture included palpation and relative applanation tonometry. Only grades II, III and IV of Baker grading scale were considered in the study.

Two plastic surgeons not involved in the study completed an evaluation questionnaire to assess aesthetic results. The panel was asked to rate the reconstructive results on a scale (visual analogical scale, VAS = 1–10), based on standardized photographs, with 1 denoting strong disagreement and 10 indicating strong agreement. The evaluation took place at least 6 months after the end of the reconstructive process (contralateral symmetrization procedures included, when performed).

## Surgical Technique

Different types of mastectomy skin incisions were performed (half-peri-areolar, inframammary fold, radial).

### Group 1

Pre-pectoral implant placement and complete coverage with ADMs comprise this cohort. Pre-shaped porcine dermis [Strattice™ (Allergan, Dublin/Ireland), Permacol™ (Medtronic, Dublin/Ireland), Braxon® (Decomed, Marcon/Italy)] or bovine pericardium tissue-derived ADMs [Veritas® (Synovis Surgical Innovations, St Paul/USA), Exaflex (Maggi Srl, Italy)] were used with an overlay tenting technique. Some of them are packed dry and need to be hydrated in saline, so they become soft after hydration. The desired implant is then placed inside the ADM. The new reconstructed ADM implant unit is placed in the pre-pectoral space and anchored to the muscular fascia using absorbable sutures.

A drain was always placed in the mastectomy pocket and another one in the axilla when axillary lymph node dissection (ALND) was performed. All the procedures were performed under general anesthesia.

### Group 2

When mastectomy had been completed, and when skin flaps were considered adequate by checking the bleeding from the edges of wounds, pectoralis major muscle dissection and detachment from chest wall were performed. Serratus muscle was spared and not used. Therefore, a retro-pectoral pocket was created. An adequately selected implant was put in place, and a biological mesh, derived from fetal and neonatal bovine dermis [SurgiMend® (Integra, Plainsboro/USA)], was then employed as a sling to cover the lower lateral pole of the implant and sutured to the pectoralis major muscle. Two suction drains were left in place: one under the combined pocket and the other in a more superficial subcutaneous site. An additional drain was employed dependent on axilla management.

Preoperative markings, including middle and parasternal lines, inframammary folds and the incision site, were executed the day before the scheduled surgery with the patient in upright position.

### Postoperative Care

All patients were discharged between postoperative day 3 and 7 after the dressing change and restraining sport-bra placement. Drainages were removed when the quantity of fluids collected was less than 30 cc after 2 days following. Patients received antibiotic therapy every 12/24 h until drain removal and were recommended to continue wearing a sport-bra for 1 month.

### Statistical Analysis

Statistical analysis was performed using R 3.3.2 (Lucent technologies, USA); *t* tests were conducted to compare the cohorts with regard to continuous variables, and Fisher’s exact tests were conducted for categorical variables. A two-tailed *p* value inferior to 0.05 was considered statistically significant.

## Results

Retrospective cohort review identified 716 consecutive mastectomies, followed by DTI breast reconstruction.

A total of 207 (28.91%) breasts underwent ADM-based implant subcutaneous placement, whereas the remaining 509 (71.09%) experienced partial muscle coverage with ADM positioned at the lower lateral pole. The timing of the reconstruction was immediate for all patients. The mean follow-up was 27.8 months for dual-plane and 16.5 months for pre-pectoral reconstruction, with a minimum of 12 months.

The two cohorts were analogous in view of oncologic characteristics, including indication for mastectomy, type of mastectomy performed and chemotherapy (Table 2).

**Table 2:** Oncologic features

Characteristic	Pre-pectoral	Dual-plane	Total	*P* value
No. of breast	207 (28.9%)	509 (71.1%)	716	
No. of patients	172 (26.8%)	470 (73.2%)	642	
Laterality				0.001
Unilateral	137 (79.7%)	431 (91.7%)	568	
Bilateral	35 (20.3%)	39 (8.3%)	74	
Mastectomy				0.0563
Nipple-sparing	147 (71.0%)	322 (63.3%)	469	
Skin-sparing	60 (29.0%)	187 (36.7%)	247	
Chemotherapy				0.2957
Neoadjuvant	20 (11.6%)	48 (10.2%)	68	
Adjuvant	41 (23.8%)	98 (20.9%)	139	
Radiotherapy				0.006
Neoadjuvant	0 (0%)	0 (0%)	0	
Adjuvant	9 (5.2%)	70 (14.9%)	79	

Seroma (4.34% in Group 1; 11.2% in Group 2. *p* value = 0.004) and hematoma (1.45% in Group 1; 4.71% in Group 2. *p* value = 0.045) were the most common postoperative complications observed, followed by surgical site infection (1.93% in Group 1, 3.93% in Group 2. *p* value = 0.2518).

Wound dehiscence rates showed no statistically significant difference between the two groups (*p* value = 0.7893) (Table 3).

As expected, we observed a significantly lower rate of animation deformity in the pre-pectoral group (Group 1) compared with partial muscle coverage group (Group 2).

Capsular contracture rate was 8.7% in the Group 1 and 13.87% in Group 2 (*p* value = 0.18180).

The difference in implant removal rates was not statistically significant between Group 1 and Group 2 (Group 1: 2.42% and Group 2: 3.93%, *p* = 0.3766). In the 1.93% and 2.94% of patients in Group 1 and Group 2, respectively, implant removal was due to its infection. We found no statistical significance in the differences of implant infection between the two cohorts (*p* > 0.05).

In the remaining cases, it was referable to implant extrusion after radiation therapy, one case in Group 1 (11.1% of irradiated breasts) and 5 cases (7.14%) in Group 2.

The overall incidence of complications was 20.77% for pre-pectoral and 32.02% for dual-plane reconstruction (*p* = 0.026).

Blinded evaluators extraneous to the study concluded that pre-pectoral reconstruction is a better aesthetic option, as scores were higher for pre-pectoral compared with dual-plane reconstructions (bilateral pre-pectoral, 8.3; unilateral pre-pectoral, 7.2; bilateral dual-plane, 6.8; and unilateral dual-plane 5.3).

Adjuvant radiotherapy was more common in Group 2, as shown in Table 2. Nonetheless, we did not find a clear correspondence between radiation therapy and the higher complication rates in this group, except for implant removal that was more common in irradiated patients.

There were no other significant differences in outcomes between the pre-pectoral and partial muscle coverage groups.

Suction drains were removed an average of 7.02 days after surgery in Group 1 and 7.09 days after surgery in Group 2, with no relevant differences between the two groups.

Results are summarized in Table 3.

**Table 3:** Comparison of postoperative complication rates between pre-pectoral and dual-plane groups

Complications	Pre–Pectoral	Dual-plane	*p* value
Seroma	9 (4.34%)	57 (11.20%)	**0**.**004**
Hematoma	3 (1.45%)	24 (4.71%)	**0**.**045**
Surgical site infections	4 (1.93%)	20 (3.93%)	0.2518
Wound dehiscence	4 (1.93%)	13 (2.55%)	0.7893
Animation deformity	0	350 (68.76%)	**0**.**00001**
Capsular contracture	18 (8.70%)	29 (13.87%)	0.1818
Implant removal	5 (2.42%)	20 (3.93%)	0.3766
**Overall complication**	43 (20.77%)	163 (32.02%)	**0**.**0026**
**Tot surgical procedures**	207	509	

Bold values indicate statistical significant (*p* < 0.05)

## Discussion

Substantial efforts have been made over the years toward the development of surgical management of breast cancer, and different techniques have been introduced in order to improve aesthetic and functional results in breast reconstruction. The use of conservative mastectomies has risen significantly during the last few years as they have been acknowledged to be oncologically safe procedures [16]. As this demand has increased so has the demand for immediate breast reconstruction. Nowadays, as we witness an increased utilization of conservative mastectomies, prosthetic breast reconstruction promotes the change from a traditional two-stage operation to a single-stage procedure [17, 18]. Historically, the widely accepted and recommended method has been sub-pectoral implant placement as it has been regarded as the pillar of implant-based reconstruction for the last 50 years. This was also due to the higher rates of major complications with subcutaneous techniques reported by early studies.

For the last 40 years, tissue expanders have been utilized [19] for a two-stage breast reconstruction in order to recover skin domain that has been lost after mastectomy. Direct-to-implant reconstruction has become more practicable with modern mastectomy techniques which can preserve increasingly larger amounts of skin, completely achieved with nipple-sparing mastectomy [20, 21]. Two-stage breast reconstruction was devised when significant skin resection was performed at the same time as mastectomy. The preferred method to restore the area of skin surface in order to insert a sufficient volume implant was expansion. Due to the adoption of newer skin- and nipple-preserving mastectomy techniques, the demand for expansion has rapidly decreased [3, 22, 23].

Several functional impairments and consequences derived from pectoralis elevation such as animation deformity and acute pain are yet unsolved. Some measure of animation deformity will be experienced by all patients with sub-pectoral implants, and this is regarded as an expected event [21–24].

The need for total sub-muscular coverage was widely substituted by the introduction and following adoption of lower pole coverage with acellular dermal matrices or synthetic meshes, in an attempt to reduce revision surgeries. Several variations to this procedure have been introduced, involving implant placement either completely or partially behind the musculature of the anterior chest wall [24–26].

The introduction of biological and synthetic meshes allowed an extension of the muscular pocket with a larger range of implantable prosthetic volume and improved aesthetic results. Nevertheless, the necessity for muscular recruitment and related patient discomfort has not been changed [14, 27–29].

The initial trend in Italian centers was to use dual-plane hybrid reconstruction partially under the muscle. However, a high level of complications has been registered.

In the meantime, pre-pectoral breast reconstruction was gaining attention in the literature due to its widespread global implementation [24, 30–32]. Therefore, it has been introduced in Italian centers and since then we have witnessed a decrease in complications and better aesthetic results.

The reason for this might be that the contraction of the pectoralis major muscle and the traction executed on the ADM at the lower lateral pole reduces the stable contact with the surrounding tissues. Without this constant contact, the ADM risks to fail in incorporating itself with the surrounding tissues that compose the implant pocket micro-environment. The lack of ADM integration into the prosthesis capsule heightens the risk of seroma, infection and implant removal.

Concomitantly, ADMs have refuted the doctrine of total muscular coverage of the prosthesis, allowing it to be positioned in part subcutaneously [3, 21, 22, 31, 33].

Pre-pectoral reconstruction has currently been revived by surgeons who have released a great surge of new techniques and preliminary reports. Several studies presented acceptable complication rates, arguing that the pre-pectoral method provides a more natural aesthetic result and avoids the concerns about raising the pectoralis major muscle, lowering postoperative pain and shortening recovery period [25, 34–37]. Despite its advantages, pre-pectoral breast reconstruction presents some limits in its application: Mastectomy flaps need to present adequate thickness and vitality, implant selection is limited to low–medium volumes, and patients with high degree of breast ptosis risk implant exposure at the inframammary fold [38].

As a consequence, the adoption of pre-pectoral reconstruction is limited to accurately selected patients. These concerns regard the failure of the device when the pectoralis muscle is absent and the postoperative aesthetics that may be compromised, including rippling and visibility of the implant [24, 39–49].

A recent article by Cattelani et al. showed increased patient satisfaction following pre-pectoral direct-to-implant reconstructions using acellular dermal matrix wrap when compared with dual-plane direct-to-implant and tissue expander/implant reconstructions [31].

At our institutions, we have observed favorable aesthetics with this approach. In the past 10 years, we have moved from sub-pectoral to pre-pectoral implant positioning improving technique and cosmetic outcomes. We are now achieving excellent results using implants fully covered by ADMs in pre-pectoral positioning avoiding a lot of procedure-related morbidity for patients [26, 50, 51].

The current study was carried out in 7 different hospitals. As a consequence, various ADMs were used, depending on the availability in every single center. Nevertheless, all the devices were considered equal in efficacy and safety terms, as it has been proved in several studies [52–54].

VAS results suggest that pre-pectoral reconstruction can give valid aesthetic outcomes even when performed unilaterally. The same cannot be said about dual-plane reconstruction. As a matter of fact, according to the questionnaires, the unilateral sub-pectoral reconstruction achieved disappointing aesthetic outcomes.

**Fig. 1:**
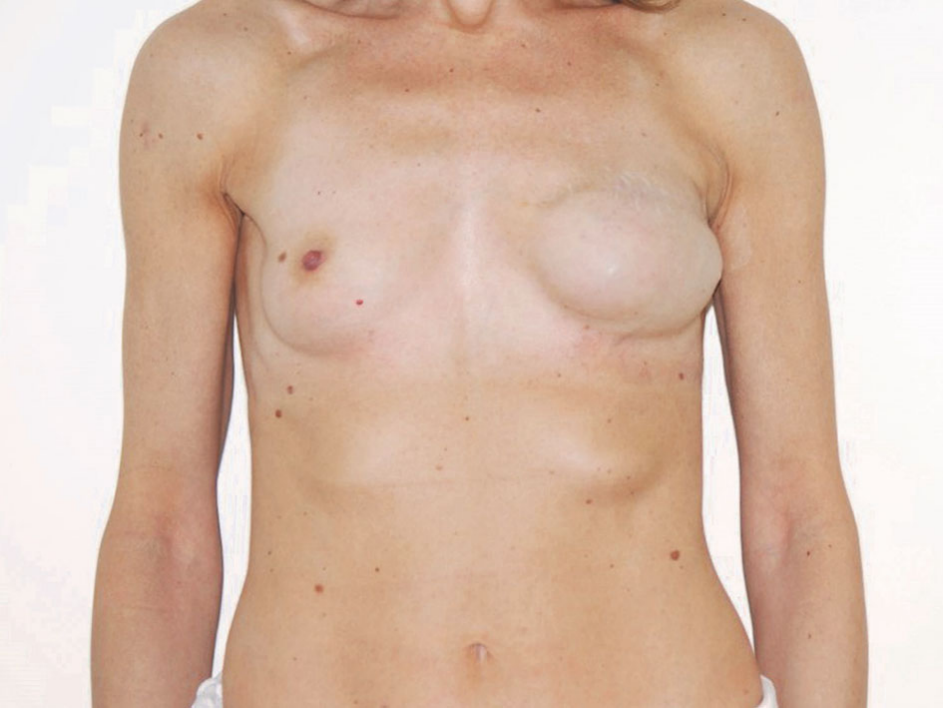
Left: Preoperative picture of a patient scheduled for right breast mastectomy. Three months earlier the patient underwent left breast mastectomy and reconstruction with temporary expander. Right: Postoperative picture of the same patient, after right breast nipple-sparing mastectomy and DTI dual-plane sub-pectoral reconstruction with ADM. In the same surgical time, left breast expander was substituted with definitive prosthesis, nipple was reconstructed with local flaps, and fat grafting was performed

**Fig. 2:**
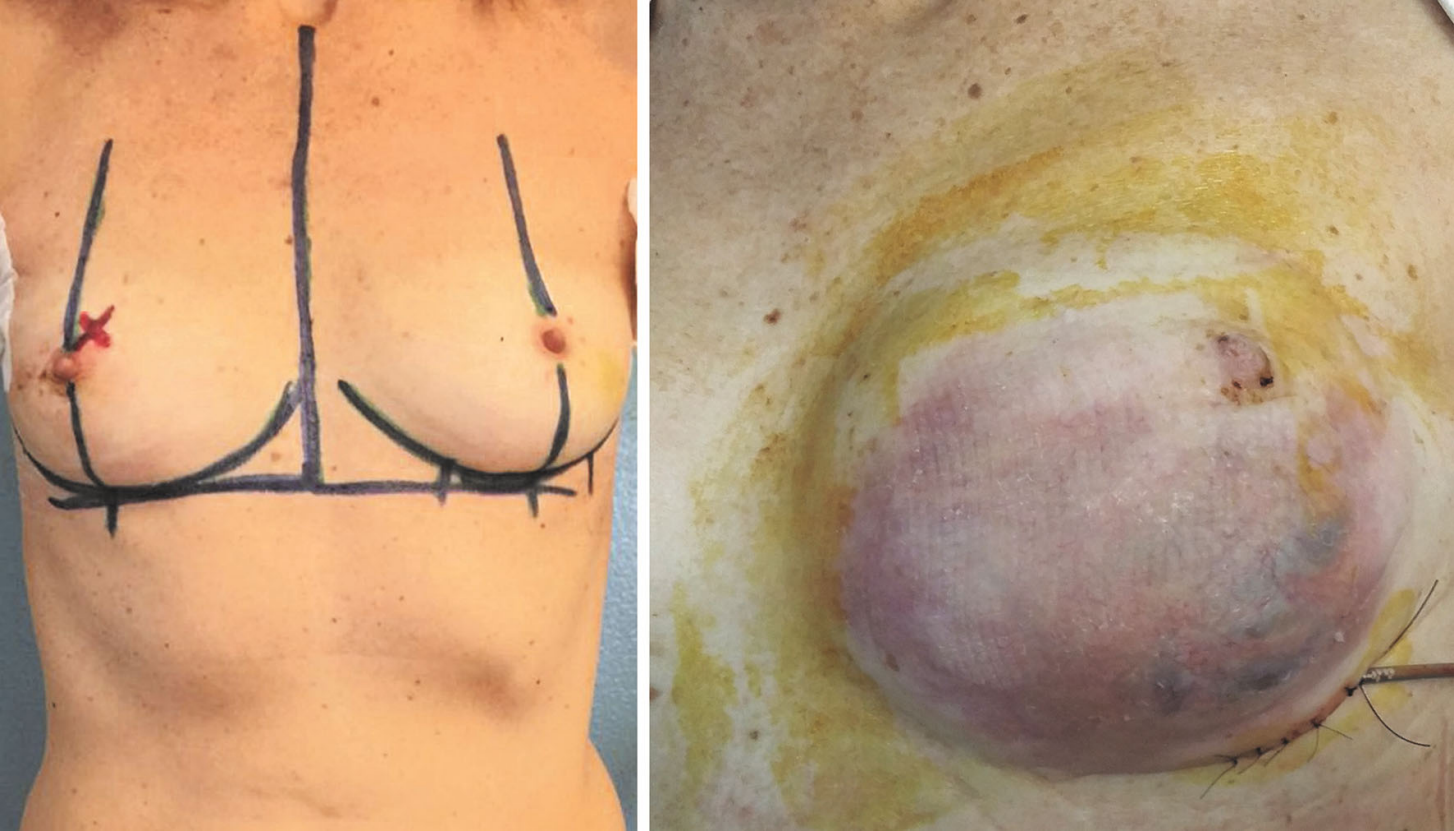
Left: Preoperative marking for bilateral mastectomy and sentinel lymph node biopsy. *Center*: The patient underwent immediate bilateral sub-muscular breast reconstruction with ADM covering the lower lateral pole of the implants. Erythema and skin suffering on the lower quadrants occurred in the left breast, which led to cutaneous necrosis, prosthesis removal and temporary expander placement. Right: Final postoperative picture after fat grafting and tissue expander replacement with permanent implant in the left breast

**Fig. 3:**
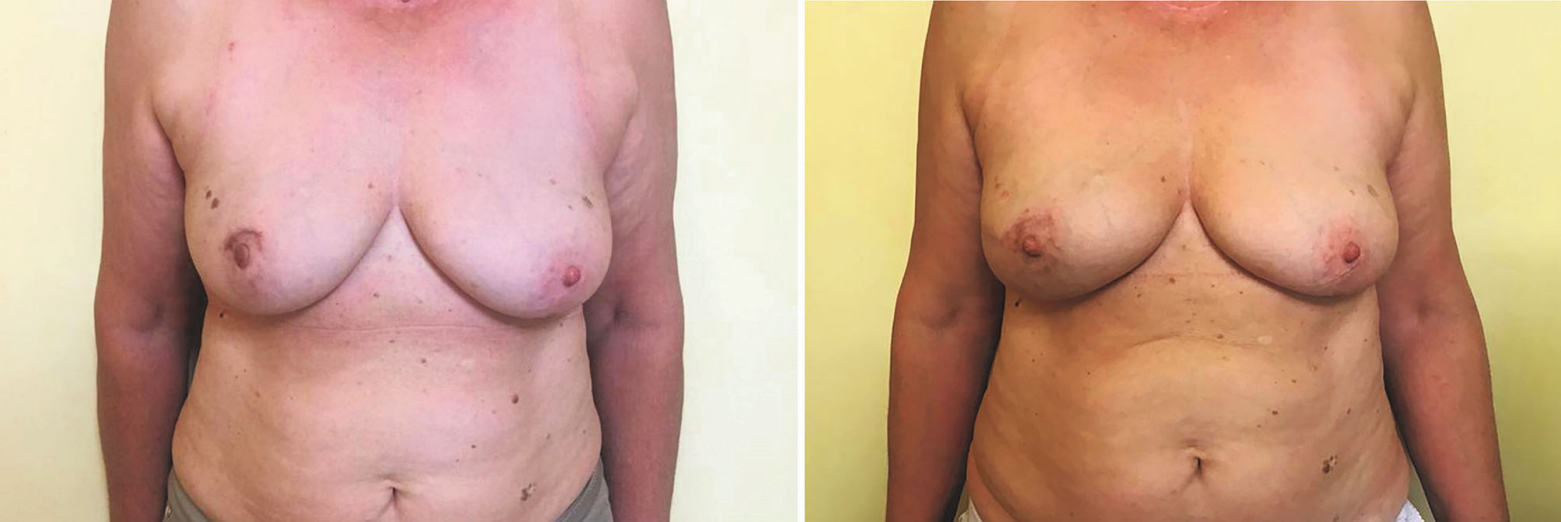
Left: Preoperative picture of a patient scheduled for right breast mastectomy. Right: Postoperative picture of the same patient, 1 year after right breast nipple-sparing mastectomy and immediate pre-pectoral reconstruction with a completely ADM-wrapped anatomical implant

A series of illustrative outcomes are demonstrated with preoperative and postoperative photographs in Figs. 1, 2 and 3.

Dynamic deformity of the implant is eliminated by pre-pectoral breast reconstruction, and the latter has the capacity to reduce postoperative pain and narcotic use, thus speeding up recovery for post-mastectomy patients when compared to the placement of sub-muscular implant [26, 55]. This objective was not reached when the dual-plane approach was used as the detachment of the pectoralis major muscle was implied [30, 31].

The majority of dual-plane reconstructions in our study was carried out before pre-pectoral reconstruction regained its popularity. Since pre-pectoral reconstruction with ADMs was introduced in our clinical practice, the reconstructive decision process switched from a preoperative decision setting toward an intraoperative one. In fact, the placement of the implant was decided according to the thickness and the vascularization of skin flaps. Thick and well-vascularized skin flaps were needed to place the implant in the pre-pectoral space to reduce any possible risk of failure. Whenever skin flaps were either not thick enough or poorly vascularized, the patient was not suitable to pre-pectoral reconstruction: In this case, a two-stage breast reconstruction with sub-muscular expander was performed.

Our study has a number of limitations worth noting, including its retrospective nature, the different length of follow-up between the two groups and the highly restrictive exclusion criteria. Also, the two cohorts are dissimilar in number of patients. Certainly, results concerning capsular contracture should be tempered by the shorter follow-up in the pre-pectoral group. Another limiting factor is the use of distinct types of ADM in each cohort that could lead to differences in complication rates and outcomes. Although one of the strengths of this study is its multicenter design, this may be one of its limitations as well. It is possible that operating protocols varied across the multiple surgeons involved in each center. Addressing both implant profile (anatomical or round) and volume was beyond the scope of this paper.

At the moment, available data on the pre-pectoral compared to dual-plane direct-to-implant approach are limited, as we are informed of only four other cohort studies which present results derived from the comparison between these two approaches [31, 39, 41, 56]. The one reported in this study represents the largest series of patients to date.

## Conclusion

Finally, we can state that retro-muscular breast reconstruction with ADM, employed to cover the lower lateral pole of the implant, brings with it the disadvantages of both retro-pectoral and pre-pectoral reconstruction. The latter, when feasible, should be considered the treatment of choice. Sub-pectoral placement of tissue expanders slightly inflated at the time of the initial surgery, remains the primary choice when plastic surgeons are confronted with thin high-risk mastectomy skin flaps. This two-stage approach minimizes the tension on the mastectomy flaps when it is clinically important to do so. At the same time, it provides the insurance of having well-vascularized muscle which protects the underlying implant in case that areas of skin flap necrosis should appear. Using the pre-pectoral approach, we have experienced superior clinical and functional outcomes with minimal pain and enhanced convenience for the patient.

Longer-term follow-up demonstrates maintenance of the integrity and quality of the reconstructions over time with low rates of capsular contracture and complete absence of animation deformity. We believe retro-pectoral muscular ADM implant coverage has to be considered only in those complicated second-stage surgeries, when the lack of soft tissues available does not allow a complete coverage of the implant, using only the thoracic muscles of the patients. An example is illustrated in Fig. 1. Strict adherence to selection criteria is mandatory to achieve optimal results.

## Electronic supplementary material

Below is the link to the electronic supplementary material.

Supplementary file1 (MOV 1943 kb)

Supplementary file2 (MP4 851 kb)
